# Estimated Number of Patients with Influenza A(H1)pdm09, or Other Viral Types, from 2010 to 2014 in Japan

**DOI:** 10.1371/journal.pone.0146520

**Published:** 2016-01-19

**Authors:** Yoshitaka Murakami, Shuji Hashimoto, Miyuki Kawado, Akiko Ohta, Kiyosu Taniguchi, Tomimasa Sunagawa, Tamano Matsui, Masaki Nagai

**Affiliations:** 1 Department of Medical Statistics, Toho University, Tokyo, Japan; 2 Department of Hygiene, Fujita Health University School of Medicine, Aichi, Japan; 3 Department of Public Health, Saitama Medical University Faculty of Medicine, Saitama, Japan; 4 Department of Pediatrics, National Mie Hospital, Mie, Japan; 5 Infectious Disease Surveillance Center, National Institute of Infectious Diseases, Tokyo, Japan; University of Calgary & ProvLab Alberta, CANADA

## Abstract

Infectious disease surveillance systems provide information crucial for protecting populations from influenza epidemics. However, few have reported the nationwide number of patients with influenza-like illness (ILI), detailing virological type. Using data from the infectious disease surveillance system in Japan, we estimated the weekly number of ILI cases by virological type, including pandemic influenza (A(H1)pdm09) and seasonal-type influenza (A(H3) and B) over a four-year period (week 36 of 2010 to week 18 of 2014). We used the reported number of influenza cases from nationwide sentinel surveillance and the proportions of virological types from infectious agents surveillance and estimated the number of cases and their 95% confidence intervals. For the 2010/11 season, influenza type A(H1)pdm09 was dominant: 6.48 million (6.33–6.63), followed by types A(H3): 4.05 million (3.90–4.21) and B: 2.84 million (2.71–2.97). In the 2011/12 season, seasonal influenza type A(H3) was dominant: 10.89 million (10.64–11.14), followed by type B: 5.54 million (5.32–5.75). In conclusion, close monitoring of the estimated number of ILI cases by virological type not only highlights the huge impact of previous influenza epidemics in Japan, it may also aid the prediction of future outbreaks, allowing for implementation of control and prevention measures.

## Introduction

Infectious disease surveillance systems provide both quantitative and qualitative information crucial for protecting populations from disastrous epidemics [1–6]. Infectious disease surveillance in Japan, initiated in 1981, has evolved into a comprehensive system of infectious disease control, particularly since the revision of the Infectious Disease Control Law in 1999 [6]. Current surveillance system in Japan, called National Epidemiological Surveillance for Infectious Diseases (NESID), which includes mandatory reporting system for national notifiable diseases and sentinel surveillance system for ubiquitous infectious diseases. National notifiable disease must require having a laboratory confirmation. Sentinel surveillance is based on disease symptom and a confirmed case distribution was reported by infectious agents surveillance, which is 10% samples of the sentinel surveillance. Specimen were randomly selected from these cases and then tested and reported by the Local Public Health Institutes. These relationships were shown in the flow diagram (S1 Appendix). Compared with other surveillance systems around the world, the Japanese system is based on a number of sentinel institutions throughout the country. In the case of influenza-like illness (ILI), almost 5000 sentinel institutions were sampled from across Japan that were representative of geographical regions (public center areas and prefectures), medical institutions (clinics/hospitals and medical departments) and population densities [7]. This carefully designed surveillance system enabled us to estimate the number of ILI patients nationwide and determine the 95% confidence intervals (95% CI) [8,9]. For example, incidence rate (per 1,000) of ILI patients in year 2005 epidemic season was estimated in 142.6 (95% CI: 135.6–149.6) [9]. This quantitative information provided important insight in to future influenza epidemics in Japan.

Identifying the virological types of influenza involved in outbreaks of infection provides essential information for the treatment and prevention of population-wide influenza epidemics. To examine the current influenza epidemic in Japan, infectious agents surveillance, which is laboratory based surveillance, investigated a small number of patients (300–600 patients in the epidemic season) and provided the distribution of virological types among patients every week. This information only reported a fraction of the virological types present among the population, but no further quantitative information was provided by the system. If the estimated number of specific virological types of influenza (i.e. pandemic influenza: A(H1)pdm09) were available, this information would help predict the threat of an influenza epidemic and therefore allow for the appropriate action to be taken.

This study estimated the number of cases of pandemic influenza (A(H1)pdm09) and seasonal-type influenza (A(H3) and B) infection from 2010 to 2014, using data provided from 5000 sentinel sites of the infectious disease surveillance system in Japan.

## Methods

### The infectious disease surveillance system in Japan

The infectious disease surveillance system in Japan comprises two types of surveillance: sentinel and infectious agents surveillance [7]. Sentinel surveillance, which covers 27 common infectious diseases including ILI, reports weekly numbers of patients throughout Japan. Sentinel medical institutions were selected by local government following the guidelines of the Ministry of Health, Labour and Welfare [10]. These guidelines were intended to ensure equal distribution of sentinel medical institutions throughout the nation. The number of sentinel medical institutions designated within each area and public health center is approximately proportional to its population size. For ILI, the total number of sentinel medical institutions in Japan is approximately 5000. ILI was confirmed by sentinel doctors (mostly using rapid diagnostic testing) of the sentinel medical institution and the number of ILI in each sentinels was reported every week using internet network system. In contrast, infectious agents surveillance is based on specimen samples, which was bought from selected sentinels (500 medical institutions). This laboratory-based surveillance confirmed the virus subtype by Local Public Health Institutes. After pathogens have been isolated from specimens, this information of local centers is sent to the central (Infectious Disease Surveillance Center) and is summarized in an infectious agents surveillance report. Together these surveillance systems provide weekly data on the frequency of infection with different virological types of influenza.

### Estimation of the number of patients with specific virological influenza types

Methods for estimating weekly numbers of ILI patients have been described previously [8,9]. In brief, estimations are based on the assumption that all sentinel medical institutions in a public health center are randomly sampled and stratified by the characteristics of the medical institutions (i.e. department type, number of patients). We used a stratified random sampling technique to estimate the total number of ILI cases and their approximate 95% confidence intervals [9].

The weekly numbers of virological type-specific patients were estimated by multiplying the above-mentioned estimated numbers of ILI with the weekly fraction of each virological type identified from the laboratory confirmed cases. The formula of this estimation is shown in the Appendix. We estimated weekly numbers of influenza cases of virological types A(H1)pdm09, A(H3) and B, from week 36 (September 6) of 2010 to week 18 (May 10) of 2014. We also conducted the age-specific estimation by using same method mentioned above. These data were also categorised according to the age of patients: 0–4, 5–19, 20–59 and 60 years and over. All statistical analyses were performed using SAS, version 9.30 (SAS Institute Inc., Cary, NC, USA).

## Results

**Fig 1:**
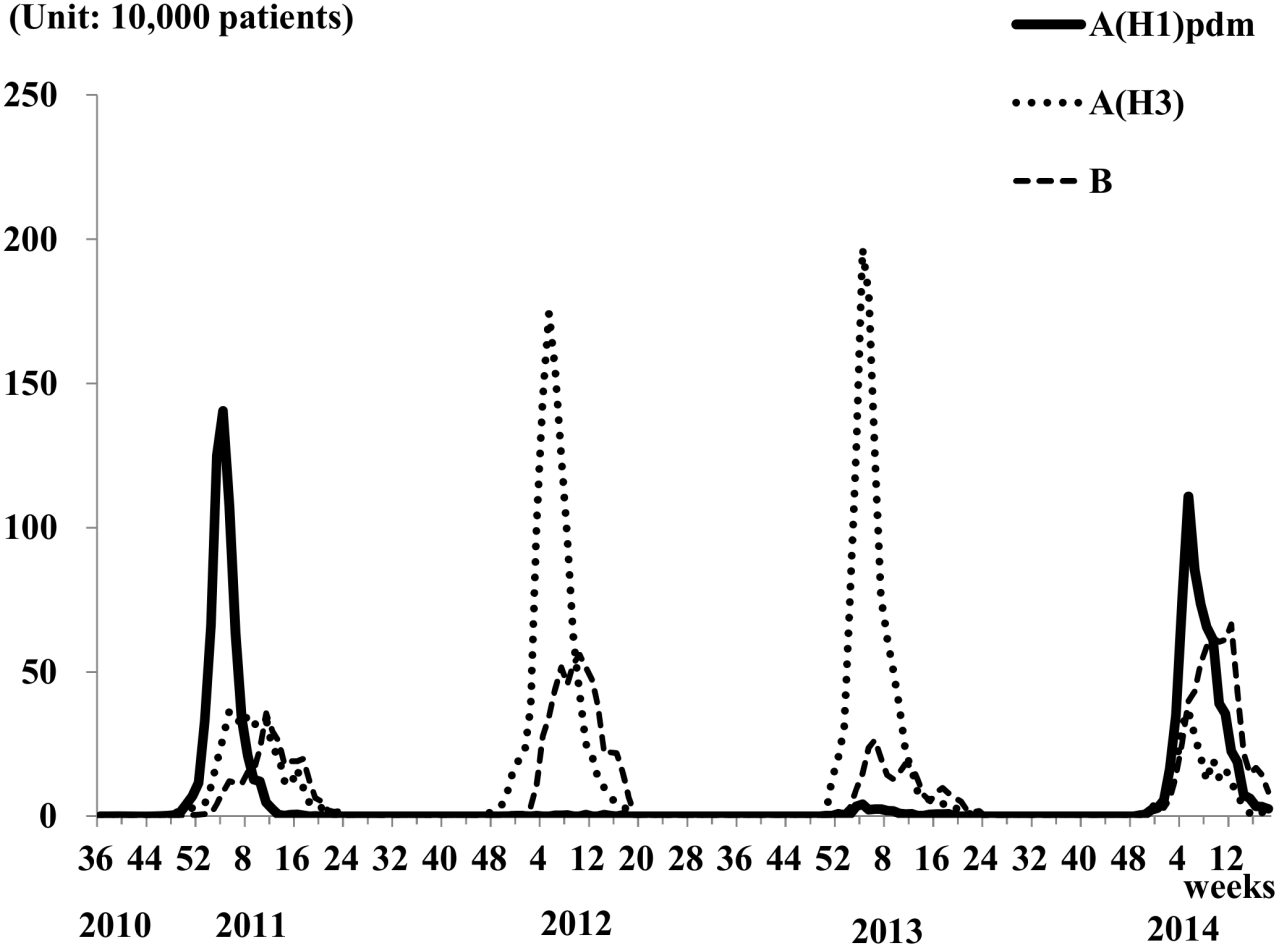
Estimated number of cases of influenza infection according to virological type from week 36, 2010 to week 18, 2014, Japan. Unit of the vertical line: 10,000 patients

Fig 1 and Table 1 show the weekly estimated number of virological type-specific influenza cases from week 36, year 2010 to week 18, year 2014, in Japan. In the 2010 epidemic season (from September 2010 to May 2011), influenza type A(H1)pdm09 was dominant [total estimated number: 6,480,000 (95% confidence interval (95% CI) 6,330,000–6,630,000]] and the largest weekly number of influenza type A(H1)pdm09 patients was observed in week 4, year 2011 [1,405,000 (1,332,000–1,477,000)], followed by seasonal influenza [A(H3): 364,000 (319,000–409,000) in week 5, B: 357,000 (307,000–407,000) in week 11] (Table 1). The incidence rates of influenza A(H1)pdm09, A(H3) and B were 0.05, 0.03 and 0.02, respectively. While in the 2011 epidemic season (from September 2011 to May 2012), seasonal type influenza A(H3) was dominant [total estimated number: 10,890,000 (10,640,000–11,140,000)], followed by influenza type B [total estimated number: 5,540,000 (5,320,000–5,750,000)]. This trend was similar in the 2012/13 season; however, in the 2013/14 season, influenza type A(H1)pdm09 was again dominant [total estimated number: 6,740,000 (6,530,000–6,950,000)].

**Table 1:** Estimated number of virological type-specific influenza-like illness patients during the 2010–2014 epidemic seasons.

	Estimated number of influenza patients (95% confidence intervals)			
	2010/11	2011/12	2012/13	2013/14
Annual total (10 thousands)				
A(H1)pdm09	648 (633–663)	3 (1–5)	26 (20–32)	674(653–695)
A(H3)	405 (390–421)	1089 (1064–1114)	1073(1053–1093)	254(239–269)
B	284 (271–297)	554(532–575)	229 (215–243)	616 (596–637)
Peak week (10 thousands)				
A(H1)pdm09	140.5 (133.2–147.7)	-	-	110.8 (102.8–118.8)
	Week 4	-	-	Week 5
A(H3)	36.4 (31.9–40.9)	174.4(164.4–184.4)	196.7(188.9–204.5)	36.4(34.4–45.2)
	Week 5	Week 5	Week 4	Week 5
B	35.7 (30.7–40.7)	55.9(49.3–62.4)	26.4(20.8–32.0)	66.5 (59.6–73.3)
	Week 11	Week 10	Week 6	Week 12

The estimated number of cases of influenza A(H1)pdm09 infection were excluded because their weekly numbers of patients were all below 5000.

**Fig 2:**
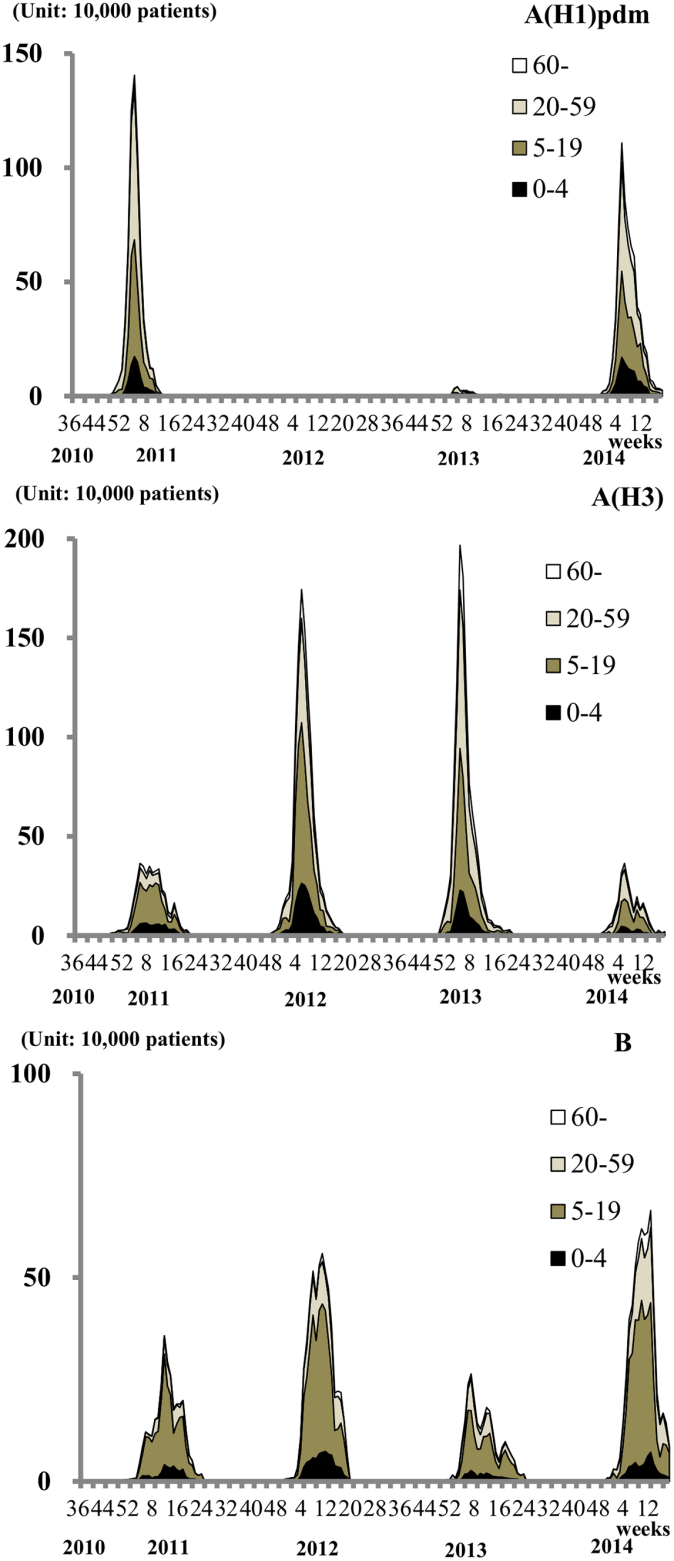
Age distribution of the estimated number of cases of influenza infection according to virological type from week 36, 2010 to week 18, 2014, Japan. Unit of the vertical line: 10,000 patients

Fig 2 shows the age distribution of patients among the estimated number of virological type-specific influenza cases during the four-year period from 2010 to 2014. During this period, approximately 50% of patients were aged 5–19 or younger, with few patients aged 60 and over, for all of the virus types. Type B influenza incidence showed a particularly strong relationship with younger age groups. For example, in 12 weeks of 2014, the number type B influenza patients of 5–19 years old was 367,000 (95% CI; 318,000–416,000), which was significantly larger than that of 20–59 years old (185,000 (95% CI; 144,000–225,000)).

## Discussion

In this study, the number of virological type-specific influenza cases over a four-year period (from week 36, 2010 to week 18, 2014) in Japan was estimated using information from the infectious disease surveillance database. An epidemic of influenza A(H1)pdm09 first occurred in Japan in 2009 [11,12]. Our estimated numbers clearly show the huge impact of influenza A(H1)pdm09 on the Japanese population in the 2010/2011 and 2013/2014 seasons.

The proposed method for the estimation of virological type-specific numbers of influenza was based on a stratified-sampling technique. This technique requires a large number of sentinel medical institutions and a well-designed protocol for selecting the location of these institutions. Infectious disease surveillance in Japan, which includes a large number of sentinel medical institutions equally distributed throughout the nation, is best suited to using a sampling technique to estimate the number of influenza patients. In the United States, the Centers for Disease Control and Prevention applied a probabilistic multiplier model for the estimation of the number of influenza A(H1)pdm09 cases nationwide [13]. In this method, a nested structure of six steps was considered for sample collection (including reported cases, test-detected cases, specimen-tested cases, specimen-collected cases, cases seeking medical care for influenza, and total influenza cases) and the multiplier (the inverse of the sampling proportion at each step) was used to estimate the total number of influenza cases [5,13]. This method is straightforward but incorporates many assumptions regarding the “multipliers” in the estimation process. Our estimation method was based on a stratified random sampling technique, the rationale for which was provided by the sampling design. Local public centers selected sentinel medical institutions in Japan based on well-designed criteria to ensure that they were distributed equally across the nation. Although the uncertainty of adherence to these criteria remained in our estimation, a large number of sentinel medical institutions (almost 5000) demonstrated pseudo-stratified random sampling. One study in Japan [14] claimed that our technique might result in an overestimation compared with other methods. This was because the sentinel medical institutions with a high frequency of patient visits were more likely to be included in the infectious disease surveillance system. Improved methods for the unbiased selection of sentinel medical institutions would therefore be desirable [15]. Data on the virological type of influenza cases were derived from laboratory-based infectious agents surveillance. This type of surveillance generated fewer samples compared with sentinel surveillance, leading to instability in the estimation of virological type-specific influenza. Confidence intervals represent the stability of an assessing estimation and the narrow range of confidence intervals shown in Table 1 demonstrate that the annual estimate of the number of virological type-specific influenza cases showed acceptable stability.

Several limitations should mentioned for our study. First, we did not present any regional-specific result that could compare the dominant sub/types of influenza in different regions. In the database of infectious disease agents surveillance, we do not have any regional-based information of influenza sub/types. We suppose that the dominant subtype of influenza in some regions might be different from those of other regions. In case that a regional specific information of influenza virus type is available, we can apply our methods for estimating the regional specific results. The enhancement of infectious disease agents surveillance is desirable to achieve that goal.

Second, we should comment on the inherent bias of our estimation. As we mentioned, the selection of sentinel medical institutions is based on the protocol. In the protocol, the sentinel doctors were encouraged to choose samples randomly, but the decision were totally depend on the doctors. So, we cannot deny the possibility of an inherent bias of specimen selection in the surveillance.

From a public health perspective, the annual estimated number of cases of influenza-like illness throughout the nation provides essential information on which to base future prevention and control strategy decisions to avoid nationwide influenza epidemics. For example, the estimated number of influenza A(H1)pdm09 cases in the 2010/11 season showed a magnitude similar to the first epidemic of this virological type of influenza in 2009 in Japan. This information is vital in preparing for future influenza epidemics [16], with preparations including vaccine storage, increasing medical facilities and provisions, and assigning a budget for ensuing costs. In our opinion, data on the weekly number of patients with virological type-specific influenza could also be made available if this estimation process was built into the infectious surveillance system. This close monitoring of infections would enable early stage intervention to help prevent future influenza epidemics.

## Supporting Information

S1 AppendixThe flow diagram of infectious disease surveillance system in Japan.Footnote; This flow diagram cited was Reference 7.**Appendix:** The formula for the point estimate and the 95% confidence intervals for the number of virological type-specific influenza-like illness cases.i is an age category and j is a category of virological type of influence-like illness. A proportion of virological type j in an age-category i sample from the virological surveillance system is *p*_*ij*_ and its variance is *v*_1*ij*_. Estimated number of cases of influenza-like illness and its variance in the age category i is *α*_*i*_ and *v*_2*i*_, which is derived from previous studies [8,9]. The estimated number of cases of influenza-like illness from virological type j in the age category i is *α*_*ij*_ = *p*_*ij*_*α*_*i*_ and its variance is expressed as $v_{ij}\;=\;v_{1ij}v_{2i}+\alpha_{i}^{2}v_{1ij}+p_{ij}^{2}v_{2i}$ [17]. Finally, the total estimated number of cases of virological type (j) -specific influenza-like illness and its variance was derived by *α*_∙j_ = Σ_*i*_*p*_*ij*_*α*_*i*_ and *v*_∙j_ = Σ_*i*_*v*_*ij*_. The approximate 95% confidence interval for α_∙∙_ is given to be $\left(\text{max}\;\left\{0,\;\alpha_{∙j}-1.96\sqrt{v_{.j}}\right\},\;\alpha_{∙j}+1.96\sqrt{v_{.j}}\right)$.(PPTX)
