# Supplementary material for: Estimated Number of Patients with Influenza A(H1)pdm09, or Other Viral Types, from 2010 to 2014 in Japan
Source: PLoS One. 2016 Jan 19;11(1):e0146520. doi: 10.1371/journal.pone.0146520 (PMC4718664; doi:10.1371/journal.pone.0146520)
Supplement: S1 Appendix — Footnote; This flow diagram cited was Reference 7. Appendix: The formula for the point estimate and the 95% confidence intervals for the number of virological type-specific influenza-like illness cases. i is an age category and j is a category of virological type of influence-like illness. A proportion of virological type j in an age-category i sample from the virological surveillance system is pij and its variance is v1ij. Estimated number of cases of influenza-like illness and its variance in the age category i is αi and v2i, which is derived from previous studies [8,9]. The estimated number of cases of influenza-like illness from virological type j in the age category i is αij = pijαi and its variance is expressed as vij=v1ijv2i+αi2v1ij+pij2v2i [17]. Finally, the total estimated number of cases of virological type (j) -specific influenza-like illness and its variance was derived by α∙j = Σipijαi and v∙j = Σivij. The approximate 95% confidence interval for α∙∙ is given to be (max0,α∙j-1.96v.j,α∙j+1.96v.j). (PPTX) [file pone.0146520.s001.pptx]

## Slide 1
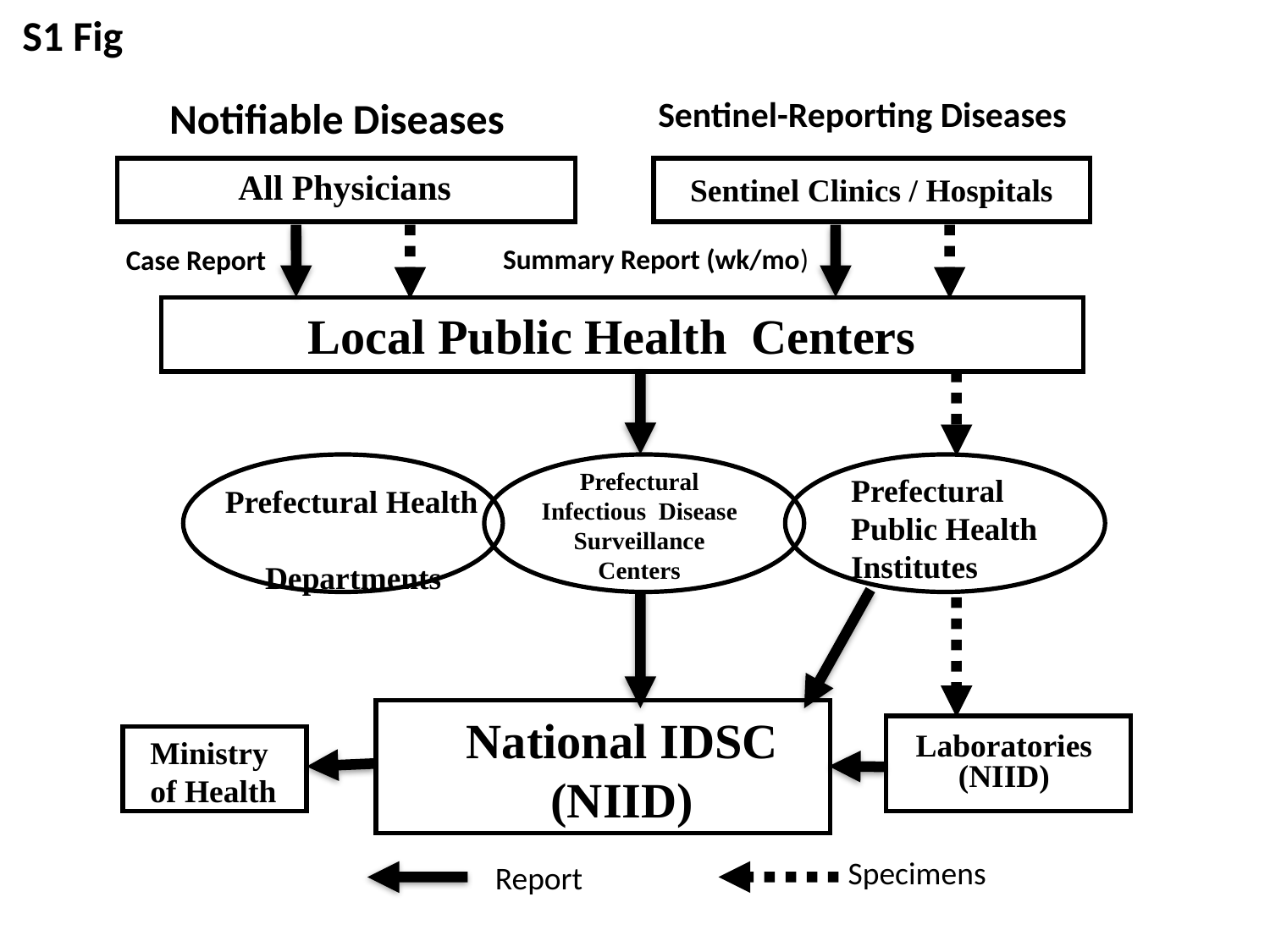

S1 Fig
Notifiable Diseases
Sentinel-Reporting Diseases
All Physicians
Sentinel Clinics / Hospitals
Summary Report (wk/mo)
Case Report
Local Public Health Centers
Prefectural Infectious Disease Surveillance Centers
Prefectural Public Health Institutes
Prefectural Health
 Departments
National IDSC
(NIID)
Ministry of Health
Laboratories (NIID)
Specimens
Report
